# Cervical epidural spinal cord stimulation is associated with enhanced voluntary upper-limb muscle activation in chronic post-stroke hemiparesis: a pilot feasibility study

**DOI:** 10.3389/fnbeh.2026.1914244

**Published:** 2026-10-26

**Authors:** Ciou-Chan Wu, Hsiang-Ling Huang, Yu-Chen Chen, Po-Kai Wang, Ming-Yung Wu, Lian-Cing Yan, Shinn-Zong Lin, Sheng-Tzung Tsai

**Affiliations:** 1 Institute of Medical Sciences, Tzu Chi University, Hualien, Taiwan; 2 Department of Neurosurgery, Hualien Tzu Chi Hospital, Buddhist Tzu Chi Medical Foundation, Hualien, Taiwan; 3 Department of Nursing, Hualien Tzu Chi Hospital, Buddhist Tzu Chi Medical Foundation, Hualien, Taiwan; 4 Department of Biomedical Sciences and Engineering, Tzu Chi University, Hualien, Taiwan; 5 Department of Medical Informatics, Tzu Chi University, Hualien, Taiwan; 6 School of Medicine, Tzu Chi University, Hualien, Taiwan; 7 Department of Anesthesiology, Hualien Tzu Chi Hospital, Buddhist Tzu Chi Medical Foundation, Hualien, Taiwan

**Keywords:** corticospinal tract, neuromodulation, spinal cord stimulation, stroke rehabilitation, upper limb hemiparesis

## Abstract

Upper-limb hemiparesis is a prevalent and often refractory sequela of stroke, and conventional rehabilitation approaches often yield limited long-term gains in the chronic phase. Cervical epidural electrical stimulation (EES) has emerged as a promising precision neurorehabilitation strategy to amplify residual corticospinal drive through contact-specific modulation of spinal interneuron excitability. However, the relationship between individual cervical motor recruitment patterns and functional recovery has not been systematically characterized in stroke survivors. Therefore, the present prospective pilot study investigated contact-specific motor mapping and the acute effects of cervical EES on voluntary upper-limb muscle activation in four participants with chronic post-stroke hemiparesis. Four adults with chronic post-stroke hemiparesis (time since stroke 6–28 months; baseline Fugl-Meyer Assessment for Upper Extremity [FMA-UE] scores 6–35) underwent bilateral percutaneous cervical epidural lead implantation spanning C3–T1. Intraoperative contact-specific motor mapping was performed through bipolar stimulation at 2 Hz, yielding individualized cervical recruitment maps that informed subsequent parameter selection. Task-related surface electromyography (EMG) was then recorded under EES-On and EES-Off conditions across six standardized upper-limb movements; EES-On/EES-Off amplitude ratios served as indices of acute stimulation effects. Clinical assessments, including the FMA-UE, Action Research Arm Test (ARAT), and Modified Ashworth Scale (MAS), were administered at baseline and 12–28 days after implantation, with EES active. Intraoperative mapping showed a level-dependent proximal-to-distal pattern of muscle recruitment consistent with cervical somatotopic organization. EES-On was associated with higher voluntary EMG amplitude in most muscles and tasks, with EES-On/EES-Off ratios of up to 11.4 in the wrist flexor during grasping. All four participants had higher FMA-UE scores at the post-implantation assessment (range +4 to +8 points), with changes in three participants falling within or above the published minimal clinically important difference range, and ARAT scores increased in all four participants (+1 to +6 points). Elbow flexor tone increased in two participants, and no serious adverse events occurred. Collectively, these preliminary findings suggest that contact-specific cervical EES, informed by individualized motor mapping, may represent a feasible adjunct intervention for upper-limb rehabilitation following chronic stroke, and provide a neurophysiological rationale for future controlled trials.

**Clinical trial registration:**

ClinicalTrials.gov, NCT05981989.

## 1 Introduction

Upper-limb impairment is among the most disabling consequences of stroke, affecting more than two-thirds of survivors and substantially limiting independence in daily activities (Lawrence et al., 2001; Ingram et al., 2021; Anwer et al., 2022; Feigin et al., 2022). Although intensive rehabilitation can promote early post-stroke functional recovery, many individuals reach a functional plateau within the first few months, with limited improvement thereafter (Stinear et al., 2007; Wei et al., 2013). Precision neurorehabilitation strategies that tailor intervention parameters to individual neurophysiological profiles represent a promising, albeit underexplored, approach to overcoming this plateau. Growing interest in this framework has driven the development of contact-specific neuromodulation techniques for post-stroke motor recovery. In this study, contact-specific neuromodulation refers to selecting, for each target movement, the electrode contact pair whose stimulation preferentially recruits the corresponding motor pools, as identified by contact-by-contact motor mapping, rather than applying a single stimulation configuration to all movements.

Epidural electrical stimulation (EES) of the spinal cord is a promising approach for restoring motor function after neurological injury. In individuals with spinal cord injury (SCI), EES of the lumbosacral cord can modulate spinal interneuron excitability and amplify residual descending commands, enabling volitional lower-limb movements that were previously absent (Harkema et al., 2011; Angeli et al., 2018; Hachmann et al., 2021). Cervical EES has been explored as a strategy to restore upper-limb function in cervical SCI, leveraging the anatomical relationship between electrode contacts and cervical myotomes to selectively recruit proximal and distal arm muscles (Lu et al., 2016).

Whether cervical EES can similarly facilitate voluntary motor output after stroke has only recently been investigated. The theoretical basis for this approach is that the spinal cord remains structurally intact in most stroke survivors, such that cervical circuits may be accessible to EES-mediated modulation even in the absence of direct spinal pathology (Pirondini et al., 2022). Powell et al. (2023) demonstrated that cervical EES acutely increased grip force and improved arm movement kinematics in two individuals with chronic post-stroke hemiparesis, with some functional improvements persisting beyond the stimulation period. A subsequent multi-participant study reported immediate improvements in strength and arm function in a cohort of seven participants with chronic post-stroke hemiparesis (de Freitas et al., 2026). Non-invasive approaches using transcutaneous cervical stimulation have similarly shown preliminary evidence of upper-limb facilitation post stroke (Zhang et al., 2024). However, contact-specific patterns of cervical motor recruitment in the stroke population and their relationship with functional outcomes have not been systematically characterized.

In this prospective pilot study, we investigated contact-specific motor mapping and the acute effects of cervical EES on voluntary upper-limb muscle activation in four participants with chronic post-stroke hemiparesis. Intraoperative bipolar stimulation was used to characterize the level-dependent patterns of muscle recruitment across cervical contacts, and task-related surface electromyography (EMG) was recorded with and without EES to quantify acute facilitation of voluntary motor output. Clinical assessments of motor function and spasticity were performed at baseline and 12–28 days after implantation, during EES combined with rehabilitation. This study addressed two exploratory research questions: first, whether intraoperative contact-specific mapping would reveal a somatotopically organized pattern of upper-limb muscle recruitment in individuals with chronic post-stroke hemiparesis, and second, whether cervical EES combined with rehabilitation would be associated with changes in standardized measures of upper-limb motor function. The primary aim was to generate preliminary neurophysiological and clinical data to characterize the feasibility and signal of effect of this approach, thereby informing the design of future controlled trials.

## 2 Materials and methods

### 2.1 Study design and participants

This prospective pilot study enrolled adults aged 20–70 years with a first-ever ischemic or hemorrhagic stroke occurring more than 6 months before enrollment and persistent upper-limb motor impairment. The exclusion criteria included major depressive disorder, dementia, cognitive impairment precluding the ability to follow verbal instructions, active infection at the planned implantation site, and fixed contracture or skeletal deformity of the upper limb that would preclude meaningful motor assessment. All participants provided written informed consent prior to their enrollment. The study was approved by the Institutional Review Board of Hualien Tzu Chi Hospital, Buddhist Tzu Chi Medical Foundation (IRB No. IRB112-125-A) and registered at ClinicalTrials.gov (NCT05981989).

**Figure 1:**
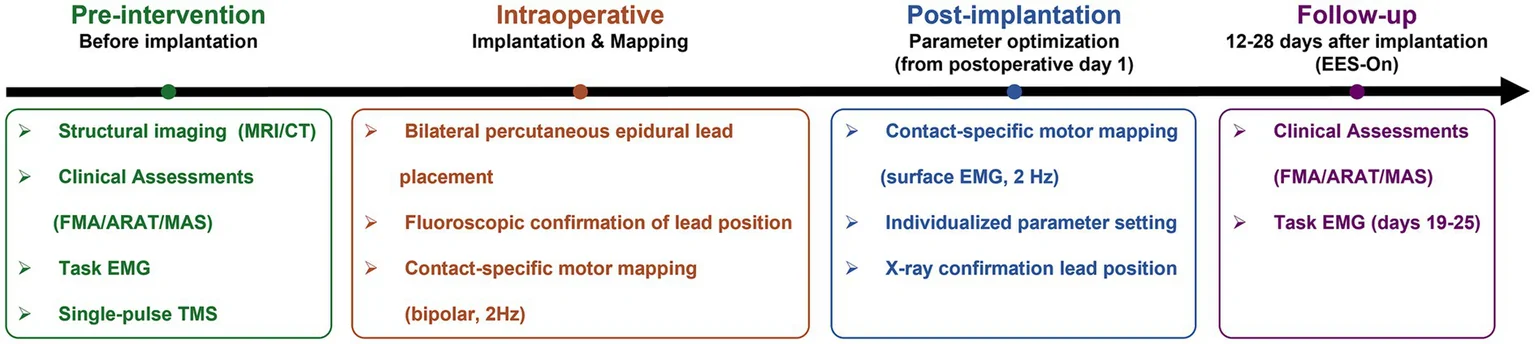
Four-stage study protocol. Pre-intervention assessments (green) were conducted before implantation (clinical assessments 1–20 days and TMS 15–38 days before implantation) and included structural imaging (MRI/CT), single-pulse transcranial magnetic stimulation neurophysiology, clinical assessments (FMA-UE, ARAT, MAS), and task-related surface EMG without stimulation, which was not analyzed in the present study. Intraoperative procedures (orange) included bilateral percutaneous cervical epidural lead implantation, fluoroscopic confirmation of lead position, and contact-specific motor mapping using bipolar stimulation at 2 Hz. Post-implantation parameter optimization (blue) involved reassessment of contact-specific motor mapping with EES and individualized parameter titration. Post-implantation assessments (purple) were performed 12–28 days after implantation with EES active; task EMG with EES on and off was recorded 19–25 days after implantation. EES, epidural electrical stimulation; EMG, electromyography; FMA-UE, Fugl-Meyer Assessment for Upper Extremity; ARAT, Action Research Arm Test; MAS, Modified Ashworth Scale.

### 2.2 Pre-operative transcranial magnetic stimulation

Single-pulse transcranial magnetic stimulation (TMS) was performed 15–38 days before implantation in all four participants. Stimuli were delivered over the ipsilesional motor cortex using a Magstim 200 monophasic stimulator and a 110-mm double-cone coil (model 4610-00; Magstim, Whitland, UK) with participants at complete rest. Peak-to-peak motor evoked potential (MEP) amplitudes were recorded from the paretic trapezius, deltoid, biceps, triceps, wrist flexor, wrist extensor and opponens pollicis, and from the non-paretic opponens pollicis (trapezius and opponens pollicis were not recorded in Participant 4), at stimulus intensities increasing in 5% steps up to 50–75% of maximal stimulator output (10 pulses per intensity). A MEP was considered present when at least 5 of 10 stimuli evoked a peak-to-peak amplitude of more than 50 μV. In Participant 4, only per-intensity summary values (mean of the four largest of 10 responses) were available; because these remained below 50 μV at all intensities, the MEP criterion could not be met in any recorded muscle. Because no MEP meeting this criterion was obtained in the hand muscles targeted by the protocol, a resting motor threshold could not be defined in the target muscle and is not reported; results are reported as the presence or absence of MEPs in each muscle within the tested intensity range. TMS was not repeated after implantation because of safety concerns regarding interaction between the magnetic field and the implanted leads.

### 2.3 Surgical procedure

Lead implantation was performed under general anesthesia. A short-acting neuromuscular blocking agent was used to facilitate endotracheal intubation and was subsequently discontinued to preserve neuromuscular transmission during intraoperative monitoring. Two percutaneous epidural leads (Octrode™, model 3086; Abbott Inc., Abbott Park, IL, USA), each with eight contacts, were advanced bilaterally into the cervical epidural space under fluoroscopic guidance to span the C3–T1 vertebral level. Lead position was confirmed intraoperatively by fluoroscopy. Postoperative MRI was performed to assess spinal cord anatomy; lead positions were subsequently co-registered onto MRI images based on intraoperative fluoroscopic coordinates, as direct lead visualization on MRI was precluded by metallic artifact (Figure 2). To minimize the risk of lead migration during the 28-day intervention period, all participants wore a cervical collar throughout the study, and lead position was monitored using cervical spine radiographs obtained on postoperative day 1 and twice weekly thereafter.

**Figure 2:**
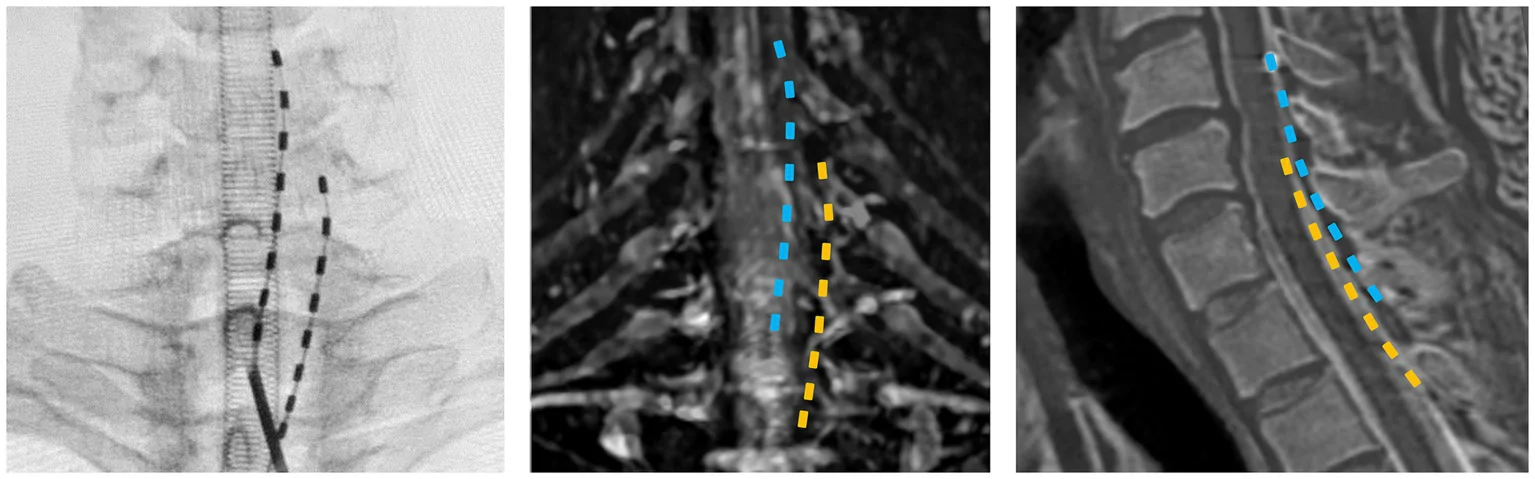
Representative imaging of cervical lead placement in Participant 2. Left: intraoperative anteroposterior fluoroscopy. Center: postoperative coronal MRI. Right: postoperative sagittal magnetic resonance imaging (MRI.) Blue and orange dashed lines indicate the two implanted leads, estimated from intraoperative fluoroscopy and superimposed on postoperative MRI for anatomical reference; direct lead visualization on MRI was not feasible, owing to metallic artifact. Leads were positioned with a rightward offset to optimize coverage of the cervical dorsal root entry zones ipsilateral to the paretic limb in this participant with right-sided hemiparesis.

### 2.4 Intraoperative contact-specific motor mapping

Following lead placement, contact-specific motor mapping was performed using needle EMG. Each contact was stimulated sequentially in a bipolar configuration at 2 Hz with pulse widths of 200–500 μs. The stimulation amplitude increased incrementally from 0.5 mA to a maximum of 5 mA or until reproducible motor responses were elicited. Peak-to-peak EMG amplitudes were recorded from upper-limb muscles selected according to participant-specific functional goals, including the deltoid, biceps brachii, triceps, flexor carpi radialis, extensor carpi radialis, extensor indicis proprius, extensor digitorum, and abductor pollicis longus. The resulting contact–muscle activation maps were used to guide the initial selection of stimulation parameters.

### 2.5 Post-implantation parameter optimization

Parameter selection followed a three-step procedure. First, the intraoperative 2-Hz contact-specific maps were used to identify, for each target muscle, the contact producing the largest peak-to-peak response, which defined the candidate cathode region. Second, on postoperative day 1, contact-specific mapping was repeated with surface EMG at 2 Hz, and bipolar configurations combining the candidate contact with adjacent contacts were tested during attempted voluntary performance of the target movement at frequencies of 10–100 Hz. Third, stimulation amplitude was increased from the sensory threshold under the direct observation of a physical therapist. The upper limit was defined as the amplitude that evoked strong involuntary contraction of the target or neighboring muscles, and therapeutic amplitudes were kept below this level so that stimulation augmented, rather than replaced, voluntary effort. The frequency and configuration producing the largest voluntary EMG amplitude in the target muscle, together with the best movement quality as judged by the attending therapist, were retained for EES-augmented therapy. Parameters were reviewed weekly based on EMG monitoring, therapist observation during task practice, and participant feedback. The intraoperative map thus defined the starting point, and the final configuration was determined by postoperative mapping and observed task performance.

Across participants, final therapeutic stimulation frequencies ranged from 20 to 100 Hz, amplitudes from 0.7 to 5.0 mA, and pulse widths from 150 to 360 μs, reflecting the degree of individualization required to match each participant’s residual motor capacity and task-specific functional goals. All therapeutic stimulations were delivered in a bipolar configuration, with cathode and anode contacts selected at spinal levels identified during intraoperative and postoperative mapping and task performance as optimal for targeting specific muscle groups. The complete parameter settings for each participant and task, together with the level of the corresponding intraoperative peak response, are provided in Supplementary Table 1; the final cathode coincided with the intraoperative peak level in 3 of 14 task configurations and lay within one segment of it in a further 6.

### 2.6 Rehabilitation protocol

All participants received conventional upper-limb rehabilitation prior to enrollment and continued their existing program throughout the study period. Rehabilitation comprised occupational therapy and physical therapy, each delivered in 30-min sessions 5 days per week (60 min per day). Sessions included task-oriented upper-limb training (e.g., picking up marbles, grasping a pen and stacking cones) and practice of the movements listed in Supplementary Table 1; content was individualized by the treating therapists according to each participant’s functional goals, whereas session frequency and duration were fixed. EES was active throughout all therapy sessions, using the configuration optimized for the movement being practiced, so that stimulation was paired with voluntary attempts at the targeted movement (Powell et al., 2023). The epidural leads remained in place for a maximum of 28 days.

### 2.7 Outcome measures

Motor function and spasticity were assessed at two time points: at baseline (1–20 days before implantation, without stimulation) and 12–28 days after implantation with EES active; post-implantation assessments were scheduled according to therapist and participant availability, and the exact interval for each participant is given in Table 1. Consistent with the intended clinical application of EES as a functional adjunct during motor activity, the post-implantation assessments were conducted with stimulation on. This design reflects real-world use, in which EES augments voluntary motor output during task performance, and the observed changes therefore represent EES-assisted performance after a short period of EES-augmented rehabilitation rather than stimulation-independent neuroplastic baseline shifts.

**Table 1:** Baseline characteristics, stimulation parameters, and outcomes of the four participants.

Characteristic/Outcome	P1	P2	P3	P4
Demographics				
Age (years)	46	27	40	46
Sex	M	M	M	M
Stroke type	Ischemic	Hemorrhagic	Hemorrhagic	Hemorrhagic
Lesion site	R Basal ganglia	L Basal ganglia	R Thalamus	L Putamen
Affected side	Left	Right	Left	Right
Time since stroke (months)	13	18	28	6
Baseline motor function				
Brunnstrom stage (proximal/distal)	III–IV/III	III/III	III/III	I/I
FMA-UE total (A–D)/66	35	21	11	6
ARAT total/57	10	3	4	0
MAS elbow flexor (pre)	1+	2	1+	0
Pre-operative MEP present	DEL, TRI	None	DEL	None
EES stimulation parameters				
Stimulation parameters	Individualized per participant and task; see Supplementary Table 1			
Outcomes post-implantation (EES-On)				
FMA-UE total (A–D)/66	43	26	15	11
ARAT total/57	13	4	7	6
MAS elbow flexor (post)	1+	3	2	0
Days from implantation to assessment	12	28	25	28

Values are presented as individual participant scores. FMA-UE, Fugl-Meyer Assessment for Upper Extremity (motor subscales A–D, maximum 66); ARAT, Action Research Arm Test (maximum 57); MAS, Modified Ashworth Scale; EES, epidural electrical stimulation; M, male; MEP, motor evoked potential (present when >50 μV in at least 5 of 10 trials); DEL, deltoid; TRI, triceps.

Assessments were performed by trained therapists and included the Fugl–Meyer Assessment for Upper Extremity (FMA-UE) (Fugl-Meyer et al., 1975), Action Research Arm Test (ARAT) (Lyle, 1981), and Modified Ashworth Scale (MAS) (Bohannon and Smith, 1987). The FMA-UE motor subscales A–D (maximum score 66) were used as the primary measure of upper-limb motor impairment. The ARAT total score (maximum 57) was used to assess functional arm and hand performance. The MAS was applied to major upper-limb muscle groups to evaluate spasticity.

### 2.8 EMG recording and analysis

Surface EMG was recorded using a Trigno Wireless System (Delsys, Inc., Natick, MA, USA), a bipolar wireless wearable EMG device. Electrodes were placed according to SENIAM guidelines for each target muscle (Hermens et al., 2000). Signals were sampled at 2,147 Hz, bandpass-filtered using a fourth-order Butterworth filter (20–450 Hz), and a 60-Hz notch filter was applied to remove power line interference. All signal processing and analyses were performed using MATLAB (MathWorks, Natick, MA, USA).

EES-On and EES-Off task EMG was recorded 19–25 days after implantation (task EMG recorded before implantation without stimulation was not analyzed) during six standardized voluntary movement tasks: shoulder abduction, shoulder flexion, elbow flexion, elbow extension, wrist flexion, and wrist extension; a grasping task was additionally recorded in Participant 1. Each task was performed five times under two conditions: EES active (EES-On) and EES inactive (EES-Off). For each trial, the signal was converted to a root-mean-square (RMS) envelope, the active-movement window was defined manually (excluding rest periods containing stimulation artifact), and the RMS envelope was integrated over this window and divided by the window duration to obtain the mean RMS amplitude (μV), so that differences in movement duration between conditions did not affect the comparison, and the mean across five trials was calculated for each condition. The ratio of EES-On to EES-Off mean amplitude was computed for each muscle and task as an index of acute EES facilitation of voluntary motor output. Absolute amplitudes are provided in Supplementary Figure 1, and representative raw EMG waveforms for each participant are provided in Figure 3.

**Figure 3:**
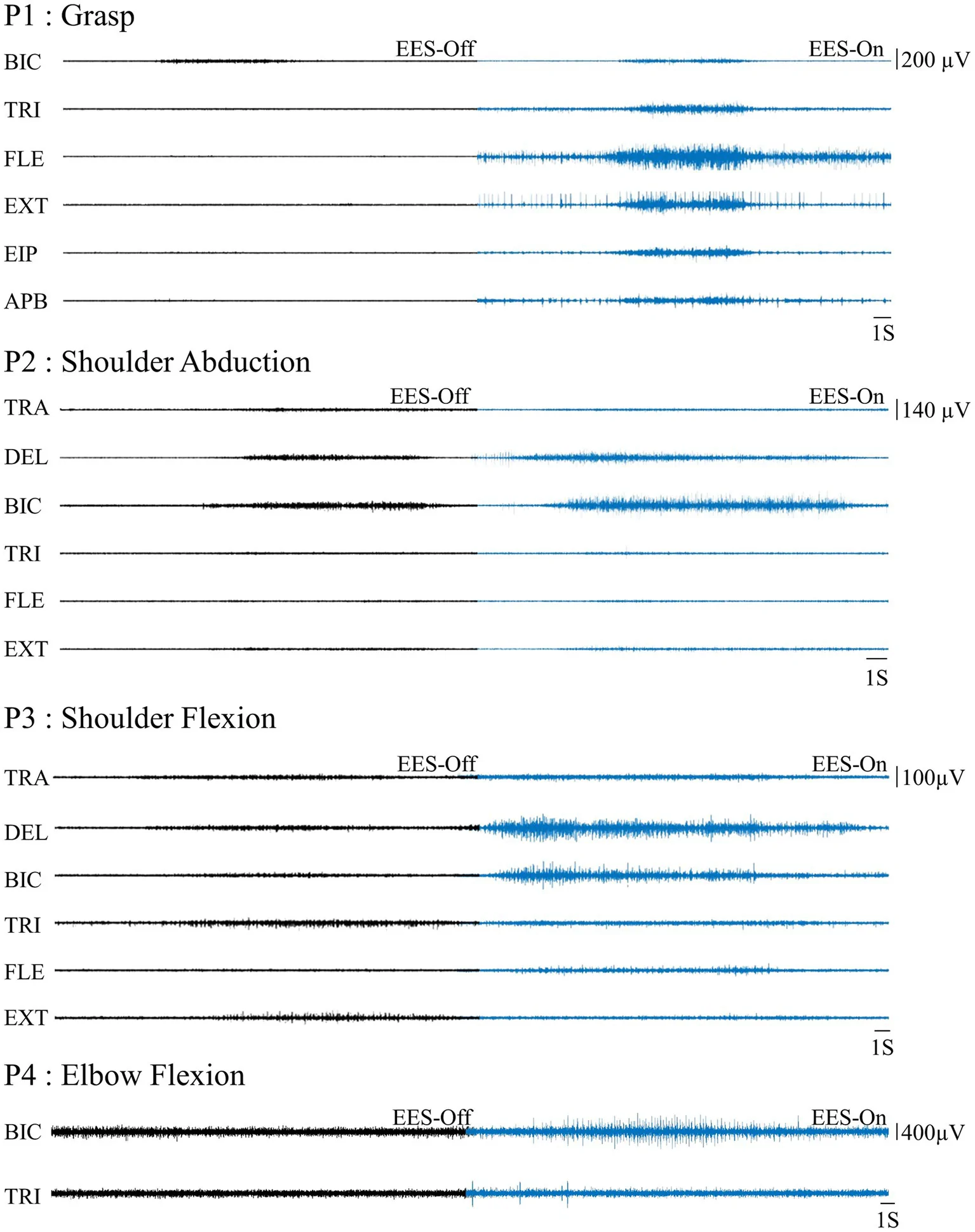
Representative surface EMG waveforms recorded during voluntary task performance in each of the four participants (P1–P4), depicted under stimulation-off (EES-Off, black) and stimulation-on (EES-On, blue) conditions. Each panel displays a single representative trial for the task yielding the highest facilitation ratio in that participant. Raw band-pass-filtered waveforms are shown. Task identity and target muscle are labeled within each panel. EES, epidural electrical stimulation; EMG, electromyography.

### 2.9 Statistical analysis

Given the exploratory nature of this pilot study and its small sample size, no formal hypothesis testing was performed. The data were presented descriptively for each participant. The EES-on/EES-off EMG ratios were reported as individual values across muscles and tasks. Clinical outcome scores (FMA-UE, ARAT, and MAS) are reported as individual scores at baseline and post-implantation, and individual change scores are summarized in Table 2. Group means and confidence intervals were not reported because, with four heterogeneous participants, such estimates would be imprecise and could imply a generalizability that the design does not support. This study was reported in accordance with the CONSORT extension for pilot and feasibility trials.

**Table 2:** Individual change scores from baseline to the post-implantation assessment.

Change score	P1	P2	P3	P4
ΔFMA-UE total (A–D)	+8	+5	+4	+5
ΔSubscale A	+1	+3	+4	+3
ΔSubscale B	+2	+2	0	0
ΔSubscale C	+5	0	0	+2
ΔSubscale D	0	0	0	0
ΔARAT total	+3	+1	+3	+6

Change = post-implantation (EES-On) score minus baseline score. FMA-UE subscales: A, upper extremity (maximum 36); B, wrist (maximum 10); C, hand (maximum 14); D, coordination/speed (maximum 6). ARAT, Action Research Arm Test (maximum 57).

## 3 Results

Four participants were enrolled and completed the study protocol (Table 1). All were male, with ages ranging from 27 to 46 years (mean, 40 years). The cohort included one participant with ischemic stroke and three with hemorrhagic stroke, with lesion sites involving the basal ganglia, thalamus, and putamen. Time since stroke ranged from 6 to 28 months, and all participants had persistent upper-limb hemiparesis meeting the chronicity criterion of >6 months.

At baseline, proximal and distal upper-limb motor function was classified as Brunnstrom stages III–IV in three participants and stage I in Participant 4, reflecting a spectrum from moderate to severe impairment (Sawner and LaVigne, 1992). Baseline FMA-UE total motor scores ranged from 6 to 35 points (out of 66), and baseline ARAT total scores ranged from 0 to 10 points (out of 57). MAS grades for elbow flexors at baseline ranged from 0 to 2 across participants.

### 3.1 Safety and feasibility

Epidural lead implantation was completed without complications in all four participants. No device-related infections, neurological deterioration, or serious adverse events were observed during the intervention period. No adverse events occurred in Participants 1–3. Participant 4 reported transient numbness during stimulation, which resolved after amplitude adjustment. No device-related infection was observed on clinical examination, serial cervical radiographs showed no lead migration, and vital signs remained normal in all participants. All participants completed the protocol, including the planned rehabilitation program.

### 3.2 Pre-operative TMS

MEPs meeting the criterion were present in the deltoid of Participants 1 and 3 and in the triceps of Participant 1. No MEP was detected in the wrist flexor, wrist extensor or opponens pollicis of any participant within the tested intensity range, and no MEP was detected in any recorded muscle in Participants 2 and 4 (Table 1).

### 3.3 Contact-specific motor mapping

Levels C3–C8 were mapped in Participant 3, whereas the other participants were mapped at a subset of levels (Supplementary Figure 2). In Participant 3, stimulation at C4–C5 produced the largest responses in proximal muscles, including the deltoid (346 μV at C4) and biceps brachii (343 μV at C4). At C6, responses shifted toward distal musculature, with the largest responses in the extensor carpi radialis (169 μV), extensor indicis proprius (118 μV), extensor digitorum (135 μV), and abductor pollicis longus (169 μV), whereas the flexor carpi radialis response peaked at C7–C8 (198–203 μV) (Figure 4A). This proximal-to-distal pattern is consistent with the expected cervical myotome distribution and suggests that individual contacts could preferentially engage different upper-limb muscle groups; given the partial level coverage in the other participants, it should be interpreted with caution.

**Figure 4:**
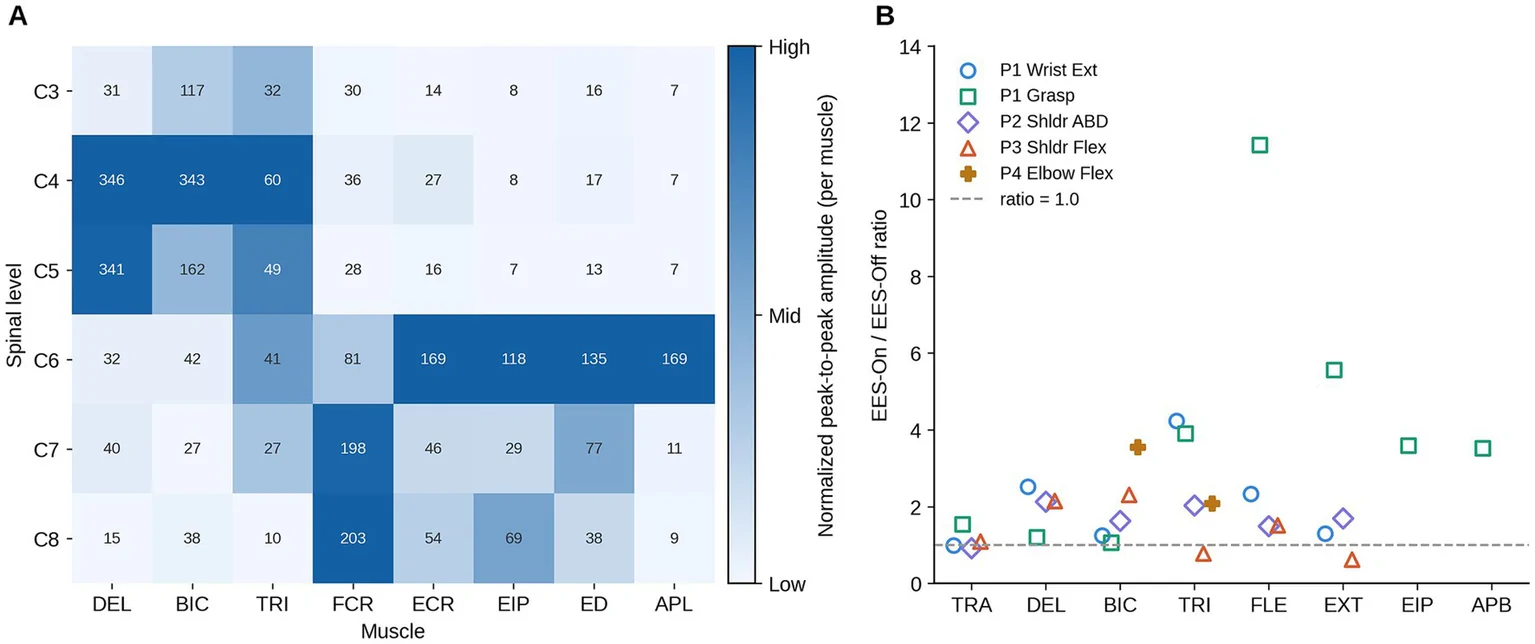
Intraoperative cervical motor mapping and acute EMG facilitation under EES. **(A)** Heatmap of mean peak-to-peak amplitude (μV) across cervical spinal levels (C3–C8) and upper-limb muscles in Participant 3, the only participant in whom all levels from C3 to C8 were mapped, during intraoperative contact-specific bipolar stimulation at 2 Hz. Cell values indicate the mean amplitude across stimulation configurations at that level; color intensity is normalized within each muscle between its lowest and highest values. Muscles: deltoid (DEL), biceps brachii (BIC), triceps brachii (TRI), flexor carpi radialis (FCR), extensor carpi radialis (ECR), extensor indicis proprius (EIP), extensor digitorum (ED), and abductor pollicis longus (APL). Proximal muscles showed maximal activation at C4–C5 and distal muscles at C6–C8. Individual maps for all participants are shown in Supplementary Figure 2. **(B)** EES-On/EES-Off EMG amplitude ratios during voluntary task performance, computed from mean RMS amplitudes within the active-movement window. Each symbol represents one participant for a specific movement–muscle combination (P1 Wrist Extension: blue circle; P1 Grasp: green square; P2 Shoulder Abduction: purple diamond; P3 Shoulder Flexion: orange triangle; P4 Elbow Flexion: brown cross). The grey dashed line denotes a ratio of 1.0. Absolute amplitudes are shown in Supplementary Figure 1. EES, epidural electrical stimulation; EMG, electromyography; TRA, trapezius; FLE, wrist flexor; EXT, wrist extensor; APB, abductor pollicis brevis.

### 3.4 Acute EES facilitation of voluntary muscle activation

During voluntary movement tasks, the EES-On condition was associated with increased EMG amplitude compared with the EES-Off condition across participants (Figure 4B; absolute amplitudes are shown in Supplementary Figure 1). The EES-On/EES-Off ratio exceeded 1.0 in 24 of 28 muscle–task combinations, consistent with facilitation of voluntary motor output, although the magnitude varied across tasks and participants.

During the grasping task in Participant 1, the wrist flexor showed the highest EES-On/EES-Off ratio (11.4; mean RMS amplitude 2.3 μV EES-Off vs. 26.3 μV EES-On), and the wrist extensor ratio during the same task was 5.6, indicating co-activation of agonist and antagonist wrist musculature under EES. In Participant 2, shoulder abduction was associated with increased activation of proximal and distal muscles, with deltoid and biceps ratios of 2.1 and 1.6, respectively. In Participant 4, elbow flexion showed a biceps ratio of 3.5. Ratios fell below 1.0 for the wrist extensor (0.62) and triceps (0.78) of Participant 3 during shoulder flexion, and were close to or below 1.0 for the trapezius of Participants 1 (0.98) and 2 (0.91). Because EES-Off amplitudes in several muscles were low (approximately 1.6–4 μV), approaching the recording noise floor, the corresponding ratios, including the highest values, are likely to overestimate the magnitude of facilitation.

### 3.5 Clinical outcomes

All four participants had higher FMA-UE total motor scores at the post-implantation assessment, with changes ranging from +4 to +8 points (Figure 5A; Table 2). Subscale analysis provided further detail on the distribution of these changes. Upper extremity motor function (subscale A) improved in all four participants (Figure 5C). Wrist motor control (subscale B) improved in Participants 1 and 2 but remained unchanged in Participants 3 and 4 (Figure 5D). Hand function (subscale C) improved in Participants 1 and 4 (from 0 to 5 points and 0 to 2 points, respectively), whereas Participants 2 and 3 showed no change in this domain (Figure 5E). Coordination and speed (subscale D) remained stable in all participants throughout the study period (Figure 5F).

**Figure 5:**
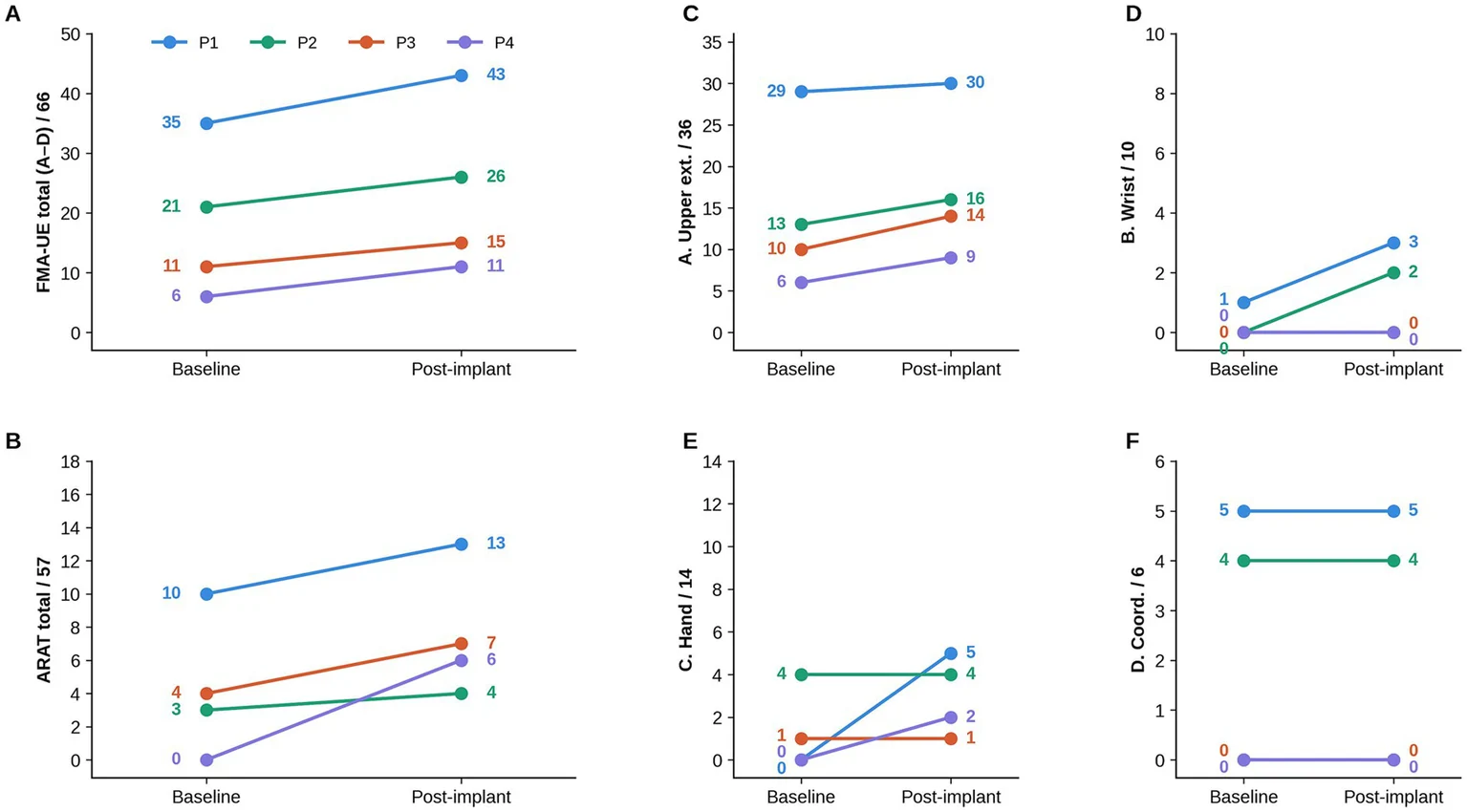
Clinical outcomes at baseline and at the post-implantation assessment (12–28 days after implantation, EES-On). Individual participant trajectories are depicted for each outcome measure (P1: blue; P2: green; P3: orange; P4: purple). **(A)** FMA-UE total motor score (subscales A–D; maximum 66). All four participants had higher scores (range +4 to +8 points). **(B)** ARAT total score (maximum 57). Scores increased in all four participants. **(C)** FMA-UE subscale A: upper extremity motor function (maximum 36). This subscale showed the most consistent change across participants. **(D)** FMA-UE subscale B: wrist motor function (maximum 10). Scores increased in Participants 1 and 2. **(E)** FMA-UE subscale C: hand motor function (maximum 14). Scores increased in Participants 1 and 4. **(F)** FMA-UE subscale D: coordination and speed (maximum 6). Scores remained stable for all participants throughout the intervention period. EES, epidural electrical stimulation; FMA-UE, Fugl-Meyer Assessment for Upper Extremity; ARAT, Action Research Arm Test.

ARAT total scores increased in all four participants (Figure 5B). Participant 4 increased from 0 to 6 points, with gains in grasp and gross movement tasks. Participants 1 and 3 each improved by 3 points, and Participant 2 showed a 1-point improvement.

Spasticity, assessed using the MAS, remained unchanged in most muscle groups; however, elbow flexor tone increased in Participant 2 (MAS 2–3) and Participant 3 (MAS 1+ to 2), and wrist flexor tone increased from 1 to 1+ in Participants 1–3. Notably, Participant 1 exhibited a baseline finger flexor MAS grade of 3, the highest recorded in the cohort; this score remained unchanged at the post-implantation assessment, indicating neither improvement nor deterioration in this severely spastic muscle group. Detailed MAS scores are provided in Supplementary Table 2.

Page et al. (2012) estimated the MCID of the FMA-UE at 4.25–7.25 points in individuals with minimal to moderate impairment due to chronic stroke. Against this range, the changes in Participants 1 (+8 points), 2 (+5 points), and 4 (+5 points) fall within or above the estimated MCID, whereas that in Participant 3 (+4 points) falls just below it. Participant 4 had severe baseline impairment (FMA-UE 6) and therefore lies outside the population in which this MCID was derived. These comparisons indicate potentially meaningful individual change and do not constitute evidence of treatment efficacy in this small, uncontrolled series.

### 3.6 Correspondence between neurophysiological and clinical findings

Changes in proximal upper extremity function (FMA-UE subscale A) were observed in all participants, in line with the consistent proximal muscle recruitment at C4–C5 in the participant with complete mapping (Figure 4A). Changes in wrist and hand function were more variable (subscale B: Participants 1 and 2; subscale C: Participants 1 and 4). Because mapping coverage differed between participants, these observations cannot be related quantitatively to individual recruitment maps.

## 4 Discussion

This pilot study examined contact-specific motor mapping and the acute neurophysiological effects of cervical EES in four individuals with chronic post-stroke hemiparesis. Intraoperative mapping revealed a level-dependent pattern of upper-limb muscle recruitment broadly consistent with the expected cervical myotome distribution. The EES-On condition was associated with increased voluntary EMG amplitude in the majority of muscles and tasks, and all participants had higher FMA-UE total scores at the post-implantation assessment during EES combined with rehabilitation. These findings provide preliminary neurophysiological and clinical evidence relevant to the development of cervical EES as an adjunct intervention for chronic upper-limb hemiparesis after stroke.

### 4.1 Cervical somatotopic organization and electromyographic facilitation patterns

The level-dependent activation pattern observed during intraoperative mapping is consistent with the somatotopic organization of the cervical spinal cord (Standring, 2021), with proximal muscles preferentially recruited at C4–C5 and distal muscles recruited at C6–C8. If confirmed in larger samples, the presence of this gradient in chronic stroke would be mechanistically relevant: unlike SCI, in which the cord itself is structurally disrupted, stroke leaves the cervical cord intact while selectively impairing descending cortical input. EES in SCI aims to activate intraspinal circuits deprived of supraspinal drive, whereas in stroke it is proposed to amplify residual corticospinal signals rather than substitute for absent ones (Pirondini et al., 2022; Powell et al., 2023). The mapping results are consistent with preserved cervical somatotopic organization after supraspinal injury, which would provide a substrate for contact-specific neuromodulation.

During voluntary movement tasks, EES-On was associated with increased EMG amplitude compared to EES-Off in most recorded muscles. This pattern is consistent with the proposed mechanism by which EES depolarizes large-diameter proprioceptive afferents in the dorsal roots, lowering the threshold for synaptic activation of spinal motor neuron pools and thereby amplifying residual descending corticospinal commands (Capogrosso et al., 2013; Hachmann et al., 2021; Pirondini et al., 2022). The magnitude of facilitation varied across muscles and tasks and did not show a consistent proximal-to-distal pattern.

In Participant 3, the wrist extensor and triceps ratios fell below 1.0 during shoulder flexion. As a post-hoc hypothesis, this response might reflect inadvertent activation of hyperexcitable flexor motoneuron pools characteristic of the post-stroke flexion synergy (Dipietro et al., 2007), rather than a global failure of EES facilitation. In chronic post-stroke spasticity, disinhibited reticulospinal pathways can contribute to abnormal co-activation and altered reflex modulation; when EES amplifies afferent drive to these hyperreflexic flexor circuits, it may paradoxically reinforce flexor-dominant patterns and suppress extensor output via reciprocal inhibitory mechanisms (Katz and Rymer, 1989). This explanation remains a post-hoc hypothesis that cannot be confirmed with surface EMG alone, but it highlights the importance of individualized parameter titration to avoid reinforcing pathological co-activation patterns.

### 4.2 Variability in clinical outcomes and changes in muscle tone

The observed improvements were lower in absolute magnitude than those reported by Powell et al. (2023) and de Freitas et al. (2026), a difference that may be partly related to baseline severity, the shorter exposure before assessment (12–28 days), and differences in protocol: our cohort had lower FMA-UE scores at entry (range, 6–35; mean, 18.3), potentially leaving less corticospinal substrate available for EES-mediated amplification. Given the absence of a control condition and the heterogeneity of the cohort in baseline impairment, lesion location, and time since stroke, the consistent direction of change should not be interpreted as evidence of efficacy. The most consistent improvement was in FMA-UE subscale A (proximal upper extremity), corresponding to the muscle groups most reliably recruited during intraoperative mapping. ARAT changes were small in three participants, and changes in hand function were limited. This dissociation is consistent with the different neural substrates captured by each assessment: the FMA-UE reflects synergistic movement patterns accessible through proximal muscle facilitation (Gracies, 2005), whereas the ARAT requires fractionated finger movements dependent on monosynaptic cortico-motoneuronal projections (Lawrence and Kuypers, 1968; Lemon, 2008; Schaechter et al., 2009; Lindenberg et al., 2010; Radlinska et al., 2010). The integrity of these projections, carried primarily by the corticospinal tract (CST), may therefore constitute a threshold below which EES-amplified spinal excitability cannot translate into coordinated distal output (Feng et al., 2015; Takase and Regenhardt, 2021). However, pre-operative TMS did not support this interpretation in our cohort: no participant showed MEPs in wrist or hand muscles, yet Participants 1 and 4 gained 5 and 2 points, respectively, on FMA-UE subscale C. The recently published feasibility trial by de Freitas et al. (2026) directly addressed this question in seven participants and found that corticospinal tract integrity, quantified by fractional anisotropy, did not predict overall FMA improvement, with spared sensory function emerging as a stronger determinant of responsiveness, though assistive effects on finger individualization remained confined to participants with residual finger movement or positive motor evoked potentials. Whether corticospinal tract integrity specifically predicts distal hand function under EES warrants further investigation in larger cohorts with comprehensive neurophysiological profiling.

The increase in elbow flexor tone in Participants 2 (MAS 2–3) and 3 (MAS 1+ to 2) warrants consideration from a safety perspective, although the MAS has limited reliability and is observer-dependent. By broadly elevating spinal excitability, EES may amplify hyperreflexic reflex circuits already disinhibited by loss of supraspinal inhibitory control (Gracies, 2005; Bhimani and Anderson, 2014; Sun et al., 2025). In Participant 3, the reduced wrist extensor and triceps ratios occurred alongside the increase in elbow flexor tone, which would be compatible with the flexion synergy hypothesis described above (Dipietro et al., 2007), in which disinhibited reticulospinal drive enhances flexor pool activity and, through spinal reciprocal inhibition (Katz and Rymer, 1989), suppresses extensor motoneuron output; however, Participant 2 showed increased tone without EMG suppression, and these observations cannot establish a mechanism. These observations preclude generalization, but it underscores the need for close muscle tone monitoring throughout EES interventions and points to the potential value of task-specific or activity-dependent closed-loop stimulation paradigms (Rowald et al., 2022) to selectively engage target circuits while limiting pathological reflex excitability.

### 4.3 Limitations

The sample size of four participants limits generalizability, and no formal statistical inference was performed. The absence of a control arm prevents isolation of EES-specific effects from those attributable to concurrent rehabilitation. As the post-implantation assessments were conducted 12–28 days after implantation and with EES active, the observed scores reflect performance under stimulation rather than a stimulation-independent neuroplastic shift; this design does not permit separation of acute facilitation from durable carry-over effects, although it captures the functional state most relevant to clinical use. Assessor blinding was not implemented, introducing the possibility of expectation bias given the criterion-referenced nature of the FMA-UE and ARAT. Diffusion tensor imaging was not obtained, TMS was performed only before implantation, only summary values were available for Participant 4, and a resting motor threshold could not be defined in the target hand muscles, limiting mechanistic interpretation of variability in treatment response; such biomarkers should be incorporated in future work, consistent with the mechanistic framework in which repeated volitional engagement under EES, rather than stimulation alone, is thought to drive synaptic plasticity in corticospinal and spinal interneuron networks (Powell et al., 2023). Force was measured in only one participant, kinematics were not collected, and a formal agonist–antagonist co-contraction index was not computed; dedicated resting recordings with EES active, EES-Off clinical outcome assessments after implantation, and post-explant follow-up were not obtained; rehabilitation content was individualized, and the number of sessions completed before assessment differed between participants. The 28-day intervention period and absence of long-term follow-up preclude conclusions regarding durability and safety; longer-term studies should monitor lead migration, infection, muscle tone, and neurological status, including after lead removal. These limitations notwithstanding, the data provide feasibility data and hypotheses to inform the design of future controlled trials, for example comparing identical rehabilitation with and without EES, or assessing performance with EES on and off at each time point and after lead removal to separate assistive from therapeutic effects.

## Data Availability

The datasets generated and/or analyzed for this study are not publicly available due to institutional data protection policies and patient confidentiality requirements, but are available from the corresponding author upon reasonable request, subject to approval by the Institutional Review Board of Hualien Tzu Chi Hospital.
