# Supplementary material for: Cervical epidural spinal cord stimulation is associated with enhanced voluntary upper-limb muscle activation in chronic post-stroke hemiparesis: a pilot feasibility study
Source: Front Behav Neurosci. 2026 Oct 26;20:1914244. doi: 10.3389/fnbeh.2026.1914244 (PMC13760448; doi:10.3389/fnbeh.2026.1914244)
Supplement: Supplementary file 1 [file Data_Sheet_1.docx]

**Supplementary Materials**

**List of Supplementary Items**

Supplementary Figure 1. Absolute EMG amplitudes under EES-On and EES-Off conditions.

Supplementary Figure 2. Individual intraoperative contact-specific motor maps for all participants.

Supplementary Table 1. Individualized stimulation parameters for each participant and task, with the level of the corresponding intraoperative peak response.

Supplementary Table 2. Detailed Modified Ashworth Scale scores at baseline and post-implantation.

Supplementary Table 3. FMA-UE subscale and ARAT subtest scores at baseline and post-implantation.

Supplementary Table 4. Item-level FMA-UE scores at baseline and post-implantation.

Supplementary Table 5. Item-level ARAT scores at baseline and post-implantation.

**EMG Recording and Analysis**

Surface EMG signals were acquired using a Trigno Wireless System (Delsys, Inc., Natick, MA, USA) with electrodes placed according to SENIAM guidelines. Signals were sampled at 2147 Hz, filtered with a fourth-order Butterworth bandpass filter at 20–450 Hz, and a 60-Hz notch filter was applied to remove power line interference. All signal processing was performed in MATLAB (MathWorks, Natick, MA). Each voluntary movement task was performed five times under EES-On and EES-Off conditions. For each trial, the signal was converted to an RMS envelope, the active-movement window was defined manually (excluding rest periods containing stimulation artifact), and the RMS envelope was integrated over this window and divided by the window duration to obtain the mean RMS amplitude, and the mean across five trials was calculated per condition. The ratio of EES-On to EES-Off mean amplitude was computed for each muscle–task combination as an index of acute EES facilitation; a ratio >1.0 indicates net facilitation of voluntary motor output under stimulation.

**Supplementary Figure 1. Absolute EMG amplitudes under EES-On and EES-Off conditions**

Mean RMS EMG amplitude (µV; integrated RMS within the active-movement window divided by the window duration, mean of five trials) under EES-Off and EES-On conditions for each muscle–task combination. Each pair of bars represents one muscle recorded during one task; colors indicate stimulation condition (grey = EES-Off, blue = EES-On). The ratio of EES-On to EES-Off amplitude is indicated above each pair. Data are presented separately for each participant (P1–P4) to preserve individual response profiles.

**Supplementary Figure 2. Individual intraoperative contact-specific motor maps for all participants**

Mean peak-to-peak amplitude (µV) at each spinal level for each recorded muscle during intraoperative contact-specific bipolar stimulation at 2 Hz, shown separately for Participants 1–4. Colour intensity is normalized within each muscle and participant between the lowest (channel noise floor) and highest values. Grey cells: level or muscle not recorded. Hatched cells: no response above noise (maximum less than twice the channel floor or less than 20 µV above it). Levels C3–C8 were mapped only in Participant 3.

**Supplementary Table 1. Individualized stimulation parameters for each participant and task, with the level of the corresponding intraoperative peak response**

| **Task** | **Cathode (spinal level)** | **Anode (spinal level)** | **Frequency (Hz)** | **Amplitude (mA)** | **Pulse width (μs)** |
| --- | --- | --- | --- | --- | --- |
| ***Participant 1*** | | | | | |
| Wrist extension (ECR; peak C8*) | C5 | C5 | 20 | 5.0 | 200 |
| Finger extension (ED; peak C6) | T1 | T1 | 50 | 5.0 | 350 |
| ***Participant 2*** | | | | | |
| Shoulder abduction (DEL; peak C5*) | C4 | C4 | 100 | 0.9 | 200 |
| Shoulder flexion (DEL; peak C5*) | C4 | C4 | 20 | 3.5 | 150 |
| Wrist extension (ECR; peak C8) | C5 | C6 | 20 | 2.8 | 300 |
| ***Participant 3*** | | | | | |
| Shoulder abduction (DEL; peak C4) | C4 | C5 | 60 | 0.9 | 200 |
| Shoulder flexion (DEL; peak C4) | C4 | C5 | 60 | 0.9 | 200 |
| Shoulder extension (DEL, TRI; peak C4) | C6 | C7 | 30 | 1.1 | 200 |
| Elbow flexion (BIC; peak C4) | C5 | C5 | 20 | 0.8 | 210 |
| Elbow extension (TRI; peak C4) | C6 | C7 | 30 | 1.1 | 200 |
| Wrist flexion (FCR; peak C8*) | T1 | T1 | 20 | 0.7 | 200 |
| Wrist extension (ECR; peak C6) | C7 | C7 | 60 | 1.4 | 200 |
| Finger extension (ED; peak C6) | C7 | C6 | 60 | 1.6 | 200 |
| ***Participant 4*** | | | | | |
| Elbow flexion (BIC; peak C5) | C5 | C6 | 60 | 2.1 | 360 |

Cathode and anode positions are indicated by the corresponding spinal level. For each task, the target muscle and the spinal level of its largest intraoperative response (peak) are given in parentheses; * indicates that the final cathode level was not covered by the intraoperative mapping in that participant. The final cathode coincided with the intraoperative peak level in 3 of 14 configurations and lay within one segment of it in a further 6; final settings were determined by postoperative mapping and therapist-observed task performance (see Methods). All stimulation was delivered in bipolar configuration. Parameters reflect the final optimized settings used during the intervention period (up to 28 days). The number of parameter configurations varied across participants according to individual functional goals and the range of voluntary movements attempted during rehabilitation. Parameters are listed only for tasks actively targeted during EES-augmented therapy sessions.

**Supplementary Table 2. Detailed Modified Ashworth Scale scores at baseline and post-implantation**

| **Muscle group** | **P1** | | **P2** | | **P3** | | **P4** | |
| --- | --- | --- | --- | --- | --- | --- | --- | --- |
|  | **Baseline** | **Post** | **Baseline** | **Post** | **Baseline** | **Post** | **Baseline** | **Post** |
| Shoulder Flexor | 1 | 0 | 1 | 1 | 1 | 1 | 0 | 0 |
| Shoulder Extensor | 1 | 0 | 1 | 1 | 1 | 1+ | 0 | 0 |
| Shoulder Abductor | 1 | 0 | 1 | 1 | 1 | 1 | 0 | 0 |
| Shoulder Adductor | 1 | 0 | 1 | 1 | 1 | 1 | 0 | 0 |
| Elbow Flexor | 1+ | 1+ | 2 | 3 | 1+ | 2 | 0 | 0 |
| Elbow Extensor | 0 | 0 | 1 | 1 | 1 | 1 | 0 | 0 |
| Wrist Flexor | 1 | 1+ | 1 | 1+ | 1 | 1+ | 0 | 0 |
| Wrist Extensor | 1 | 1 | 1 | 1 | 1 | 1 | 0 | 0 |
| Fingers Flexor | 3 | 3 | 2 | 2 | 1+ | 1+ | 0 | 0 |
| Fingers Extensor | 1 | 0 | 1 | 1+ | 1 | 1 | 0 | 0 |

MAS scores: 0 = no increase in muscle tone; 1 = slight increase; 1+ = slight increase with catch; 2 = more marked increase; 3 = considerable increase. All scores reflect the paretic upper limb.

**Supplementary Table 3. FMA-UE subscale and ARAT subtest scores at baseline and post-implantation**

| **Assessment** | **P1** | | **P2** | | **P3** | | **P4** | |
| --- | --- | --- | --- | --- | --- | --- | --- | --- |
| **Assessment** | **Baseline** | **Post** | **Baseline** | **Post** | **Baseline** | **Post** | **Baseline** | **Post** |
| ***FMA-UE (Motor Subscales A–D, max 66)*** | | | | | | | | |
| Subscale A – Upper extremity (max 36) | 29 | 30 | 13 | 16 | 10 | 14 | 6 | 9 |
| Subscale B – Wrist (max 10) | 1 | 3 | 0 | 2 | 0 | 0 | 0 | 0 |
| Subscale C – Hand (max 14) | 0 | 5 | 4 | 4 | 1 | 1 | 0 | 2 |
| Subscale D – Coordination/speed (max 6) | 5 | 5 | 4 | 4 | 0 | 0 | 0 | 0 |
| Total (A–D, max 66) | 35 | 43 | 21 | 26 | 11 | 15 | 6 | 11 |
| ***ARAT (max 57)*** | | | | | | | | |
| Grasp (max 18) | 2 | 3 | 2 | 2 | 1 | 3 | 0 | 3 |
| Grip (max 12) | 2 | 2 | 0 | 1 | 2 | 3 | 0 | 0 |
| Pinch (max 18) | 0 | 1 | 0 | 0 | 0 | 0 | 0 | 0 |
| Gross movement (max 9) | 6 | 7 | 1 | 1 | 1 | 1 | 0 | 3 |
| Total (max 57) | 10 | 13 | 3 | 4 | 4 | 7 | 0 | 6 |

FMA-UE, Fugl–Meyer Assessment for Upper Extremity; ARAT, Action Research Arm Test; P = Participant. Post-implantation assessments were performed with EES active, 12–28 days after implantation.

**Supplementary Table 4. Item-level FMA-UE motor scores at baseline and post-implantation**

| **Item** | **P1** | | **P2** | | **P3** | | **P4** | |
| --- | --- | --- | --- | --- | --- | --- | --- | --- |
|  | **Base** | **Post** | **Base** | **Post** | **Base** | **Post** | **Base** | **Post** |
| ***A. Upper extremity (max 36)*** | | | | | | | | |
| ***I. Reflex activity*** | ***4*** | ***4*** | ***4*** | ***4*** | ***4*** | ***4*** | ***4*** | ***4*** |
| Flexors: biceps and finger flexors | 2 | 2 | 2 | 2 | 2 | 2 | 2 | 2 |
| Extensors: triceps | 2 | 2 | 2 | 2 | 2 | 2 | 2 | 2 |
| ***II. Volitional movement within synergies*** | ***16*** | ***16*** | ***8*** | ***11*** | ***5*** | ***8*** | ***2*** | ***5*** |
| Flexor synergy: shoulder retraction | 2 | 2 | 1 | 1 | 0 | 0 | 0 | 1 |
| Flexor synergy: shoulder elevation | 2 | 2 | 2 | 2 | 1 | 1 | 1 | 1 |
| Flexor synergy: shoulder abduction | 2 | 2 | 1 | 1 | 0 | 1 | 0 | 0 |
| Flexor synergy: shoulder external rotation | 2 | 2 | 1 | 1 | 1 | 1 | 1 | 0 |
| Flexor synergy: elbow flexion | 2 | 2 | 2 | 1 | 1 | 1 | 0 | 1 |
| Flexor synergy: forearm supination | 1 | 1 | 0 | 2 | 0 | 1 | 0 | 0 |
| Extensor synergy: shoulder adduction/internal rotation | 2 | 2 | 0 | 1 | 1 | 1 | 0 | 1 |
| Extensor synergy: elbow extension | 2 | 2 | 0 | 1 | 1 | 1 | 0 | 1 |
| Extensor synergy: forearm pronation | 1 | 1 | 1 | 1 | 0 | 1 | 0 | 0 |
| ***III. Volitional movement mixing synergies*** | ***5*** | ***5*** | ***1*** | ***1*** | ***1*** | ***2*** | ***0*** | ***0*** |
| Hand to lumbar spine | 2 | 2 | 0 | 0 | 0 | 0 | 0 | 0 |
| Shoulder flexion 0–90° | 2 | 2 | 0 | 0 | 0 | 1 | 0 | 0 |
| Pronation/supination (elbow 90°) | 1 | 1 | 1 | 1 | 1 | 1 | 0 | 0 |
| ***IV. Volitional movement with little or no synergy*** | ***4*** | ***5*** | ***0*** | ***0*** | ***0*** | ***0*** | ***0*** | ***0*** |
| Shoulder abduction 0–90° | 2 | 2 | 0 | 0 | 0 | 0 | 0 | 0 |
| Shoulder flexion 90–180° | 2 | 2 | 0 | 0 | 0 | 0 | 0 | 0 |
| Pronation/supination (elbow 0°) | 0 | 1 | 0 | 0 | 0 | 0 | 0 | 0 |
| ***V. Normal reflex activity*** | ***0*** | ***0*** | ***0*** | ***0*** | ***0*** | ***0*** | ***0*** | ***0*** |
| Biceps, triceps, finger flexors | 0 | 0 | 0 | 0 | 0 | 0 | 0 | 0 |
| **Subtotal A** | **29** | **30** | **13** | **16** | **10** | **14** | **6** | **9** |
| ***B. Wrist (max 10)*** | | | | | | | | |
| Stability at 15° dorsiflexion (elbow 90°) | 1 | 1 | 0 | 1 | 0 | 0 | 0 | 0 |
| Repeated dorsiflexion/volar flexion (elbow 90°) | 0 | 1 | 0 | 0 | 0 | 0 | 0 | 0 |
| Stability at 15° dorsiflexion (elbow 0°) | 0 | 1 | 0 | 1 | 0 | 0 | 0 | 0 |
| Repeated dorsiflexion/volar flexion (elbow 0°) | 0 | 0 | 0 | 0 | 0 | 0 | 0 | 0 |
| Circumduction | 0 | 0 | 0 | 0 | 0 | 0 | 0 | 0 |
| **Subtotal B** | **1** | **3** | **0** | **2** | **0** | **0** | **0** | **0** |
| ***C. Hand (max 14)*** | | | | | | | | |
| Mass flexion | 0 | 2 | 2 | 2 | 1 | 1 | 0 | 1 |
| Mass extension | 0 | 1 | 0 | 0 | 0 | 0 | 0 | 0 |
| Hook grasp | 0 | 0 | 0 | 0 | 0 | 0 | 0 | 1 |
| Thumb adduction | 0 | 1 | 1 | 1 | 0 | 0 | 0 | 0 |
| Pincer grasp, opposition | 0 | 0 | 0 | 0 | 0 | 0 | 0 | 0 |
| Cylinder grasp | 0 | 1 | 1 | 1 | 0 | 0 | 0 | 0 |
| Spherical grasp | 0 | 0 | 0 | 0 | 0 | 0 | 0 | 0 |
| **Subtotal C** | **0** | **5** | **4** | **4** | **1** | **1** | **0** | **2** |
| ***D. Coordination/speed (max 6)*** | | | | | | | | |
| Tremor | 2 | 2 | 2 | 2 | 0 | 0 | 0 | 0 |
| Dysmetria | 2 | 2 | 2 | 2 | 0 | 0 | 0 | 0 |
| Time | 1 | 1 | 0 | 0 | 0 | 0 | 0 | 0 |
| **Subtotal D** | **5** | **5** | **4** | **4** | **0** | **0** | **0** | **0** |
| **Total A–D (max 66)** | **35** | **43** | **21** | **26** | **11** | **15** | **6** | **11** |

Scores: 0 = cannot perform; 1 = performs partially; 2 = performs fully. Base, baseline (without stimulation); Post, post-implantation assessment with EES active (12–28 days after implantation). Italic rows give subsection totals. FMA-UE, Fugl–Meyer Assessment for Upper Extremity.

**Supplementary Table 5. Item-level ARAT scores at baseline and post-implantation**

| **Item** | **P1** | | **P2** | | **P3** | | **P4** | |
| --- | --- | --- | --- | --- | --- | --- | --- | --- |
|  | **Base** | **Post** | **Base** | **Post** | **Base** | **Post** | **Base** | **Post** |
| ***Grasp (max 18)*** | | | | | | | | |
| Block, 10 cm cube | 0 | 0 | 0 | 0 | 0 | 0 | 0 | 0 |
| Block, 2.5 cm cube | 1 | 1 | 0 | 0 | 0 | 1 | 0 | 1 |
| Block, 5 cm cube | 0 | 1 | 1 | 1 | 1 | 1 | 0 | 1 |
| Block, 7.5 cm cube | 0 | 0 | 1 | 1 | 0 | 1 | 0 | 1 |
| Cricket ball | 1 | 1 | 0 | 0 | 0 | 0 | 0 | 0 |
| Sharpening stone | 0 | 0 | 0 | 0 | 0 | 0 | 0 | 0 |
| **Subtotal** | **2** | **3** | **2** | **2** | **1** | **3** | **0** | **3** |
| ***Grip (max 12)*** | | | | | | | | |
| Pour water from glass to glass | 0 | 0 | 0 | 0 | 1 | 1 | 0 | 0 |
| Tube, 2.25 cm | 1 | 1 | 0 | 1 | 1 | 1 | 0 | 0 |
| Tube, 1 cm | 1 | 1 | 0 | 0 | 0 | 1 | 0 | 0 |
| Washer over bolt | 0 | 0 | 0 | 0 | 0 | 0 | 0 | 0 |
| **Subtotal** | **2** | **2** | **0** | **1** | **2** | **3** | **0** | **0** |
| ***Pinch (max 18)*** | | | | | | | | |
| Ball bearing, thumb–ring finger | 0 | 0 | 0 | 0 | 0 | 0 | 0 | 0 |
| Marble, thumb–index finger | 0 | 1 | 0 | 0 | 0 | 0 | 0 | 0 |
| Ball bearing, thumb–middle finger | 0 | 0 | 0 | 0 | 0 | 0 | 0 | 0 |
| Ball bearing, thumb–index finger | 0 | 0 | 0 | 0 | 0 | 0 | 0 | 0 |
| Marble, thumb–ring finger | 0 | 0 | 0 | 0 | 0 | 0 | 0 | 0 |
| Marble, thumb–middle finger | 0 | 0 | 0 | 0 | 0 | 0 | 0 | 0 |
| **Subtotal** | **0** | **1** | **0** | **0** | **0** | **0** | **0** | **0** |
| ***Gross movement (max 9)*** | | | | | | | | |
| Hand behind head | 2 | 2 | 0 | 0 | 0 | 0 | 0 | 1 |
| Hand on top of head | 2 | 2 | 0 | 0 | 0 | 0 | 0 | 1 |
| Hand to mouth | 2 | 3 | 1 | 1 | 1 | 1 | 0 | 1 |
| **Subtotal** | **6** | **7** | **1** | **1** | **1** | **1** | **0** | **3** |
| **Total (max 57)** | **10** | **13** | **3** | **4** | **4** | **7** | **0** | **6** |

Scores: 0 = cannot perform any part of the test; 1 = performs partially; 2 = completes but takes abnormally long or has great difficulty; 3 = performs normally. Base, baseline (without stimulation); Post, post-implantation assessment with EES active (12–28 days after implantation). ARAT, Action Research Arm Test.
